# Relationship Between Medical Students’ Empathy and Occupation Expectation: Mediating Roles of Resilience and Subjective Well-Being

**DOI:** 10.3389/fpsyg.2021.708342

**Published:** 2021-09-27

**Authors:** Wenzhi Wu, Qiqi Qi, Xin Cao, Shujun Li, Zhichao Guo, Lei Yu, Xiao Ma, Yilin Liu, Zijun Liu, Xu You, Yatang Chen, Qing Long, Zhaowei Teng, Yong Zeng

**Affiliations:** ^1^The Sixth Affiliated Hospital of Kunming Medical University, Yuxi, China; ^2^School of Maxism, Kunming Medical University, Kunming, China

**Keywords:** empathy, resilience, subjective well-being, medical students, occupation expectation

## Abstract

**Background:** The occupation expectation of medical students can predict the possibility of their future employment in the medical industry, and empathy is the special ability of medical students in their study and career, which affects the development of their occupation expectation.

**Objective:** To explore the relationship between resilience and subjective well-being between medical students’ empathy and occupation expectation and their internal mechanisms.

**Design:** Data were collected from October 2020 to March 2021 using a paper questionnaire survey.

**Subjective:** 586 medical students at a key medical university in Yunnan Province were invited to complete the survey.

**Main Measures:** The Basic Empathy Scale, Connor-Davidson Resilience Scale, Subjective Well-Being Questionnaire, and Occupation Expectation Scale.

**Key Results:** The empathy is intended to affect the occupation expectation of medical students through four paths. The direct path effect value is 0.073 (95% CI: 0.007∼0.217), the indirect path 1 effect value is 0.078 (95% CI: 0.022∼0.134), indirect path 2 effect value is 0.010 (95% CI: 0.005∼0.022), indirect path 3 effect value is 0.022 (95% CI: 0.0604∼0.039), all the confidence intervals do not contain 0, and the mediated effect ratio is 60.109%.

**Conclusion:** Empathy has an impact on occupation expectation of medical students through the sequential mediating effects of resilience and subjective well-being. Medical colleges should fully consider the role of protective factors when cultivating and enhancing the occupation expectation of clinical medical students. Strengthening the intervention of emotional factors (empathy), self-regulating ability (psychological toughness) and cognitive factors (subjective well-being) is an important way to effectively establish professional values, improve occupation expectation of medical students and reduce the turnover rate of medical students.

## Introduction

A study from *The Lancet* analyzed that 4.7 million medical graduates were trained in China from 2005 and 2015, while the total number of doctors increased by only 750,000, and the turnover rate was as high as 84%. The proportion of doctors aged 25 to 34 fell from 31.3 to 22.6% over a 10-year period, and the gap in rural doctors in China is currently as high as 500,000 (Lien et al., 2016). What causes medical students to study medicine but do not practice medicine?

Occupation expectation is the belief of the ultimate state or behavior that people can obtain from a certain occupation, the evaluation of people’s social professional needs, and the reflection of life values on professional problems. The occupation expectations of medical students indicate their desire or yearning for the profession of doctor, which is not only the external expression of the intrinsic professional values of medical students, but also the internal driving force that determines the individual’s career choice (Wu and Li, 2001). The college period is a critical period for medical students to constantly understand themselves and make “trying professional decisions” and form occupation expectation, and also affect their future intention to pursue a career as a doctor. The self-concept theory of Super’s career development opinion suggests that college students are in the exploration period of the formation of self-employment concept. Their career choice is in the transition state of ideal and reality, and if they can guide it correctly and effectively, then college graduates can correctly match their occupation expectation with the social value of their actual profession (Gable and Pruzek, 2015). Therefore, it is necessary to pay attention to the formation and development of occupation expectation of medical students in the university study stage, so as to enhance the intention of medical students to engage in medical work after graduation. Health care workers need to understand the patient’s experiences and ideas and use them as a basis for communicating with patients in order to help them relieve their pain (Hojat, 2009), which requires their empathy ability. Studies have shown that the cultivating the empathy ability not only plays an important role in maintaining the perfect personality, promoting physical and mental health and improving professional quality, but also helps to ease the tension relationship between doctors and patients and improve the doctors’ sense of achievement in the future medical practice (Xie et al., 2017), and the sense of professional achievement and prestige status are important factors that affect occupation expectation (Wu and Li, 2001). However, the influence mechanism of medical students’ empathy on their occupation expectation still needs to be further explored. The Relational Development System (RDS) theory holds that individuals can achieve the best match with their situation through self-regulation mechanisms such as resilience, so as to obtain high satisfaction and promote their positive development (Guo et al., 2017). Therefore, resilience may integrate emotional factors (empathy) and cognitive factors (subjective well-being) in the formation and development of medical students’ occupation expectations. In conclusion, in order to further clarify the mechanism of empathy on occupation expectations of medical students, it is necessary to investigate the influence mechanism of emotional factors (empathy), self-regulation ability (resilience) and cognitive factors (subjective well-being) on their occupation expectations from the perspective of multi factor integration, with a view to providing theoretical basis for improving medical students’ occupation expectation and reducing the brain drain of medical personnel.

### The Impact of Empathy on Occupation Expectation

Empathy is “an individual’s emotional response based on an understanding of another person’s emotional state or condition, which is equivalent to or similar to what others are experiencing or may experience” (Eisenberg and Miller, 1987). Emotional empathy and cognitive empathy are the two main components of empathy, the former refers to the emotional response to other people’s emotions, which means the generation of emotional experiences similar to others, and the latter refers to understanding the causes of other people’s emotional state (Fan et al., 2011; Miklikowska et al., 2011; Smith, 2017). Some studies have pointed out that the total score of the empathy ability is negatively related to the emotional tiredness of job burnout and work achievement (Qi et al., 2011), and the violence in the workplace would reduce the empathy ability and seriously affect the level of job burnout (Li et al., 2016). The empathy ability has a very important role to play in maintaining a good doctor-patient relationship (Kestenbaum et al., 1989; Sherman and Cramer, 2005), and is related to personal well-being gained in the work (Shanafelt et al., 2005). A study of 18 studies on the empathy ability of medical students and clinicians shows that there are many complex reasons behind the decline in the empathy ability in medical education and clinical professionalism, including job burnout, low well-being, and quality of life reduced (Neumann et al., 2011). Empathy can help medical staff prevent work stress (Jorge et al., 2017), improve the quality of professional life (Laverdière et al., 2019), and then improve individual professional identity (Mao, 2019). And there are some research evidence having proved that the empathy ability can be cultivated (Batt Rawden et al., 2013). Therefore, it is an important foundation to promote the cultivation of professional spirit and form good occupation expectation to help students establish great occupation ability in the medical student stage.

Therefore, we propose the hypothesis1: Medical students’ empathy ability can significantly predict the level of occupation expectation.

### Mediating Role of Resilience

Resilience refers to an individual’s ability to respond to traumatic events, which is the ability to maintain relative stabile and healthy level of behavior and physical activity, and the ability to generate experience and positive emotions (Scali et al., 2012). Studies have shown that individual’s resilience is predicting their occupation expectation (He et al., 2019), and has a significant positive correlation with professional maturity. Individuals with high levels of resilience are able to develop better coping mechanisms to manage stress when faced with work difficulties, thereby learning to overcome job burnout (Wang and Song, 2011), improve occupation expectations, and reduce willingness to leave (Matos et al., 2010). In addition, the researchers find that resilience negatively predicts job burnout and subjective employment barriers (Zhang and Jin, 2010), and has a significant negative correlation with anxiety about choosing a career (Zhao K. et al., 2013).

Meanwhile, resilience, as an internal psychological trait, is affected by the individual’s empathy ability. Kinman and Grant’s research finds that emotional intelligence (including the empathy ability) can promotes resilience (Kinman and Grant, 2010), while resilience is influenced by the empathy ability (Morice-Ramat et al., 2018). The framework of resilience in action points out that the innate potential of individuals is the source of resilience, and external protective factors from society schools, families, relatives and peers can help young people develop individual internal resources, including empathy, cooperation, problem solving and so on, which will protect young people from risk factors and promote their healthy development (Li and Zhang, 2006). The integrated factor-process model of resilience also shows that the improvement of resilience comes from the interaction of environmental predictors, subject characteristics and negative life events, while the empathy, as a subject feature involving behavior/society, is the necessary condition for individuals to overcome external pressures and challenges form environmental prediction factors and to promote physical and mental system reform in the process of resilience (Kumpfer and Bluth, 2004; Xi et al., 2013). Previous studies have found that resilience acts as an mediation between empathy and depression (Shi et al., 2021). The type of attachment of individuals with high empathy is more in favor of the safety type, and their resilience are also higher (Qi et al., 2019). Medical students with high empathy are able to stand from the point of view of others, perceiving more emotional and practical support, and can actively adjust the response to maintain the love and expectations of the doctor’s profession in the face of difficulties (Shi et al., 2021).

Therefore, we propose the hypothesis2: Medical students’ empathy is positively correlated with resilience, which in turn is positively correlated with occupation expectation. In other words, resilience mediates the link between Medical students’ empathy and occupation expectation.

### Mediating Role of Subjective Well-Being

Subjective well-being is defined as an individual’s overall perception and judgment of his or her quality of life according to his subjective criteria, which is subjective, stable and holistic (Diener et al., 1999), plays an important role in the formation and development of individual occupation expectation (Chen and Zhou, 2003). Subjective well-being is a multi-layered and multi-dimensional structure consisting mainly of life satisfaction and emotional experience including positive emotion and negative emotion (Wu, 2000). Life satisfaction is an overall cognitive judgment of quality of life, and the balance of positive and negative emotions reflects an individual’s emotional experience of life (Diener and Ryan, 2009). The research shows that the subjective well-being and personal growth initiative of college students show a significant positive correlation with professional identity, and have good predictive effect on each other. The subjective well-being is significantly negatively associated with the tendency to leave (Liu et al., 2014), which suggests that the willingness of medical students to switch careers may stem from a lower level of well-being from doctor’s profession. People with high levels of empathy have greater subjective well-being and higher life satisfaction (Wei et al., 2011; Bourgault et al., 2015), have more positive and less negative emotions and lower prevalence of depression (Gruhn et al., 2008; Wei et al., 2011; Wang and Zhang, 2012). In addition, the clinical practice of the empathy technology also come to the same conclusion that the use of the empathy can significantly improve the patient’s subjective well-being and promote their rehabilitation (Fang, 2017; Xiao, 2017). The expectation theory of subjective well-being holds that the individual’s evaluation of subjective well-being is always relative to certain criteria, that is, the individual’s expectation goal. If the goal is achieved, the value of subjective well-being is high and the opposite is low (Diener and Fujita, 1995). Medical students with higher empathy are more likely to perceive the pain relief and psychological comfort that a doctor can bring to patients in medical practice, and thus more able to meet their psychological expectations of becoming a doctor, such as saving lives and benefiting the society, so that they can achieve higher happiness and job satisfaction, and further strengthen their own occupation expectations meanwhile. Therefore, the promotion of occupation expectations from the empathy ability of medical students may be achieved by enhancing the subjective well-being of individuals.

Therefore, we propose the hypothesis3: Medical students’ empathy is positively correlated with subjective well-being, which in turn is positively correlated with occupation expectation. In other words, subjective well-being mediates the link between Medical students’ empathy and occupation expectation.

### Multiple Mediating Effects of Resilience and Subjective Well-Being

Individuals with high resilience tend to make more positive and optimistic assessments and expectations about the impact that problems may have on themselves. Subjective well-being is also the result of an individual’s evaluation and expectation of the impact that certain problems may have on himself, which has a certain relationship with resilience (Han et al., 2016; Li and Li, 2016). Some scholars have shown that the individuals with strong resilience have the better ability to regulate the surrounding environment (Xu, 2000) and have higher life satisfaction. Moreover, studies have shown that resilience is significantly related to subjective well-being, and that resilience can significantly predict subjective well-being, which can directly predict their mental health (Wang and Wang, 2013; Zhao J. et al., 2013; Huang and Xie, 2016; Jiao, 2020). Increasing of resilience means growth, health, and well-being, and improving resilience requires people to continuously learn and master a range of key skills from childhood to adulthood to develop a strong ability to self-regulate. Individuals with higher self-regulating ability tend to have better resilience, still believe that they have the ability to adjust their emotional state and have good emotional stability even in adversity. These people tend to change the environment, can better cope with all kinds of problems, and maintain a positive and optimistic attitude toward life, so it is possible to produce higher life satisfaction and subjective well-being. Although it is also used as a mediating factor through which the empathy affects the occupation expectations of medical students, there are no studies that have discussed the mediating mechanism of combination of resilience as a self-regulating factor and subjective well-being as a cognitive factor.

**FIGURE 1:**
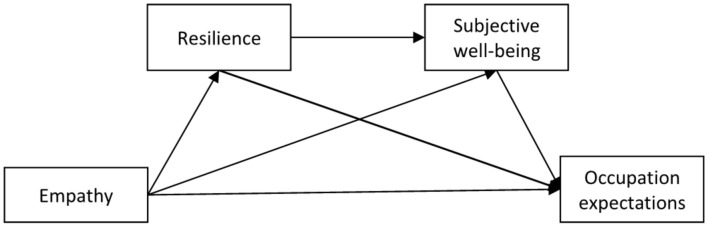
A hypothetical sequential mediating model diagram.

Therefore, we propose the hypothesis4: Resilience positively predicts subjective well-being. Thus, the association between medical student’s empathy and occupation expectation is sequentially mediated by resilience and subjective well-being. Resilience and subjective well-being may work together in a mixed mediation model involving both parallel and sequential mediating effects. In other words, resilience and subjective well-being exert parallel and sequential mediation effects on the link between medical student’s empathy and occupation expectation. A multiple mediation model (Figure 1) was established for this study in order to test all possible mechanisms of these two mediators.

## Materials and Methods

### Participants

The present study was conducted on November 2020 within one mouth. The trained graduate students majoring in psychology were selected as the main test to explain the confidentiality principle and guidelines to the participants, and make sure that they understand the questionnaire correctly. The anonymous, self-report questionnaire in Chinese was distributed to a cluster random sample of 650 Chinese college medical students who volunteered and gave informed consent. Each of the students completed all questionnaire items independently in approximately 15 min. A 609 questionnaires were collected after completing the measurement and the recovery rate was 93.692%. The final number of valid questionnaires was 586 after excluding questionnaires which had some of the incomplete key variables answer, all consistent answers or obvious laws of false answer, and the effective rate was 96.223%. The final 586 valid subjects (224 male and 362 female) were between 17 and 26 years of age (*M* = 19.898 years, *SD* = 1.547 years). Other demographic characteristics of the sample were shown in Table 1.

**TABLE 1:** Sociodemographic characteristics of the sample (*n* = 586).

Age (years, *M/SD*)	19.898 (1.547)
Gender (*n/%*)	
Boys	224 (38.2)
Girls	362 (61.8)
Grade (*n/%*)	
Freshman	150 (25.5)
Sophomore	290 (49.3)
Junior	90 (15.3)
Senior	12 (2)
Five-grade	10 (1.7)
Graduate student	36 (6.1)
Academic record (*n/%*)	
A	16 (2.7)
B	100 (17)
C	414 (70.4)
D	36 (6.1)
E	22 (3.7)
Student cadre (*n/%*)	
Yes	124 (21.1)
No	464 (78.9)
Only child (*n/%*)	
Yes	176 (29.9)
No	412 (70.1)
Family income level (monthly, *n/%*)	
Up to 4,000¥	192 (32.7)
4,000–8,000¥	192 (32.7)
8,000–12,000¥	138 (23.5)
12,000–16,000¥	30 (5.1)
More than 16,000¥	24 (4.1)
No information	12 (2)

To test the adequacy of the sample size, the present study used G^∗^Power 3.1 Software for *Post hoc* Statistical Efficacy Test (effect size = 0.15, α = 0.05), the results showed power = 1, indicating sufficient sample size (Faul et al., 2009).

The questionnaire and methodology for this study was approved by the Human Research Ethics committee of the Kunming Medical University (Ethics approval number: 2021kmykdx6f65).

### Measurement

#### Basic Information Questionnaire

According to the purpose of the study, the Basic Information Questionnaire of the subjects is compiled including the gender, age, grade, socio-economic status of the family and so on.

#### Basic Empathy Scale

The Basic Empathy Scale consists of 16 items and measures two aspects of empathy abilities: (1) cognitive empathy, (2) emotional empathy. The questionnaire uses a five-point Likert scale ranging from 1 (completely disagree) to 5 (completely agree). Responses across the 16 items were summed to obtain the total score, with higher scores indicating higher capacity for empathy (Jolliffe and Farrington, 2006). Studies have proved that it has good reliability and validity in the context of Chinese culture (Rong et al., 2021). In this study, the second-order CFA model generated a very good fit, with χ*2/df* = 2.171, *p* < 0.001, *RMSEA* = 0.071, *GFI* = 0.910, *AGFI* = 0.860, *IFI* = 0.857, *CFI* = 0.850, and *PGFI* = 0.582, and both the absolute and value-added adaptation indexes were in the acceptable range. The Cronbach’s alpha coefficient for the Basic Empathy Scale was 0.827.

#### Connor-Davidson Resilience Scale

The Connor-Davidson Resilience Scale consists of 25 items and measures three aspects of resilience: (1) tenacity, (2) strength, and (3) optimism. The questionnaire uses a five-point Likert scale ranging from 1 (completely disagree) to 5 (completely agree). Responses across the 25 items were summed to obtain the total score, with higher scores indicating higher capacity for resilience (Yu and Zhang, 2007). Studies have proved that it has good reliability and validity in the context of Chinese culture (Yang et al., 2019). In this study, the second-order CFA model generated a very good fit, with χ*2/df* = 2.655, *p* < 0.001, *RMSEA* = 0.055, *GFI* = 0.934, *AGFI* = 0.909, *IFI* = 0.912, *CFI* = 0.911, and *PGFI* = 0.678, and both the absolute and value-added adaptation indexes were in the acceptable range. The Cronbach’s alpha coefficient for the Connor-Davidson Resilience Scale was 0.912.

#### Subjective Well-Being Questionnaire

The Subjective Well-Being Questionnaire was compiled by Diener including life satisfaction (5 items), positive affect frequency (6 items), and negative affect frequency (8 items) (Diener, 1984). All three scales are scored by 7 points. The total score of subjective well-being is calculated by adding positive emotions to life satisfaction minus negative emotions. Studies have proved that it has good reliability and validity in the context of Chinese culture (Yang et al., 2020). In this study, the Cronbach’s alpha coefficients of three weight scales were 0.793, 0.825, and 0.899.

#### Occupation Expectation Scale

The Occupation Expectation Scale consists of 21 items and measures three aspects of occupation expectation: (1) Prestige status and stability, (2) Intro-Value, and (3) External Value. The scale uses a five-point Likert scale ranging from 1 (completely disagree) to 5 (completely agree). Responses across the 21 items were summed to obtain the total score, with higher scores indicating higher level for occupation expectation (Wu and Li, 2001). Studies have proved that it has good reliability and validity in the context of Chinese culture (Tong et al., 2019). In this study, the second-order CFA model generated a very good fit, with χ*2/df* = 2.655, *p* < 0.001, *RMSEA* = 0.055, *GFI* = 0.934, *AGFI* = 0.909, *IFI* = 0.912, *CFI* = 0.911, and *PGFI* = 0.678, and both the absolute and value-added adaptation indexes were in the acceptable range. The Cronbach’s alpha coefficient for the Connor-Davidson Resilience Scale was 0.935.

### Statistical and Analyses

All data collected in this study were recorded on a computer and processed using SPSS 19.0 and Mplus 7.0. Data processing was carried out in three steps according to recent literature on multiple mediation analyses (Jia et al., 2017). First, a factor analysis was used to conduct a common variance analysis for testing common method biases. Secondly, scores from the four questionnaires were analyzed using descriptive statistics and correlation analysis. Finally, Mplus 7.0 was used to evaluate a multiple mediation model for the roles of resilience and subjective well-being in the link between empathy and occupation expectation (Wen and Ye, 2014).

In the specific data processing process, there are some missing values in the study due to subjects’ filling in and answering, data entry and other reasons. The study uses the Little test with completely random missing values to investigate the randomness of missing values. The results show that χ^2^ (6342) = 14127.266, *p* < 0.001, indicating that the missing value is not completely random. Therefore, we use Mean Adjusted Maximum Likelihood Estimator (MLM) to deal with missing values. In addition, this study uses χ^2^/*df*, *RMSEA, TLI, CFI*, and *SRMR* are used to evaluate the fitting of the model χ^2^/*df* less than 5, *CFI* and *TLI* greater than 0.90, *RMSEA* and *SRMR* less than 0.08 were used as the criteria for evaluating the good fitting data of the model (Schafer and Graham, 2002).

## Results

### Multicollinearity Assessment and Common Method Deviation Test

The tolerance range of all prediction variables is 0.755 ∼ 0.975 (≤0.1 means multicollinearity), and the variance inflation factor is 1.026 ∼ 1.324 (≥10 means multicollinearity), indicating that there is no multicollinearity problem among prediction variables.

The common method deviation is controlled by anonymity, different measurement forms and scoring methods, and two methods are used to test the common method deviation. (1) Common variance analysis was applied to the four questionnaires through factor analysis. The chi-square statistic of Bartlett’s test of sphericity was significant. After principal component analysis, 18 eigenvalues greater than 1 were extracted. The first factor to explain the variance was 19.860%, which was less than the 40% required by the critical standard (Jia et al., 2017), demonstrating that the questionnaires used in the current study had no significant issue with common method biases. (2) The four factor model composed of four latent variables and their indicators was tested, and the results showed that the four factor model fits well (χ^2^ = 161.608, *df* = 38, *CFI* = 0.951, *NFI* = 0.938, *RMSEA* = 0.074). Then, the single method latent factor method is adopted (Zhou and Long, 2004; Xiong et al., 2013) tested the five factor model after adding method factors, and the fitting indexes were good (χ^2^ = 75.352, *df* = 27, *CFI* = 0.981, *NFI* = 0.971, *RMSEA* = 0.055). The comparison between the four factor model and the five factor model showed that the Δχ^2^ was significant (Δ*df* = 11, Δχ^2^ = 86.256, *p* < 0.001), but Δχ^2^ would be systematically affected by the sample size, so the model comparison still needs to refer to the changes of other fitting indexes (Wen et al., 2004). It can be seen that the degree of improvement before and after *CFI*, *NFI*, and *RMSEA* was no more than 0.05, which indicated that the model fitting has not been significantly improved after adding common method factors (Li et al., 2013). Based on the results of the above two analysis methods, it could be determined that the homologous variance problem had not had a serious impact on this study.

### Descriptive Statistics and Correlation Analysis

Descriptive statistics and correlation matrix of empathy, resilience, subjective well-being, and occupation expectation are provided in Table 2. Results of bivariate correlation analysis showed that all variables were significantly and positively correlated with each other.

**TABLE 2:** Descriptive statistics and correlation matrix of all variables (*n* = 586).

Variables	*M*	*SD*	1	2	3	4
1. Empathy	3.584	0.484	1			
2. Resilience	2.539	0.454	0.135**	1		
3. Subjective well-being	5.729	2.189	0.142**	0.488**	1	
4. Occupation expectations	3.584	0.664	0.242**	0.442**	0.391*	1

**Correlation is significant at the 0.01 level (two-tailed), *Correlation is significant at the 0.05 level (two-tailed).

### Multiple Mediation Model

**FIGURE 2:**
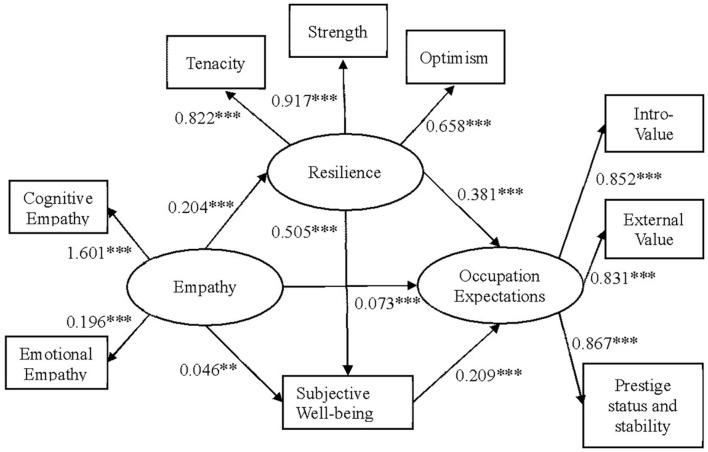
Multiple mediation model. Path values are the path coefficients. ^∗∗∗^Correlation is significant at the 0.001 level (two-tailed), ^∗∗^correlation is significant at the 0.01 level (two-tailed).

According to the theoretical hypothesis, a multiple mediation model is established in this study. Among them, empathy is independent variable, occupation expectation is dependent variable. Consider all one-way paths, resilience and subjective well-being are the mediated variables to establish the whole path model M_0_ (see Figure 2) and perform path analysis. In order to test the hypothesis model and determine the relationship between the two mediated variables, the competitive model M_1_ and M_2_ are proposed based on the hypothesis model M_0_. Competitive model M_1_ modifies the chain mediation path to “Empathy → Resilience → Subjective Well-being → Occupation Expectation.” The competitive model M_2_ deletes the path from resilience to subjective well-being constituting a parallel Multiple mediation model.

Fitness index of model M_0_ is shown in Table 3, and model M_0_ fits well. The path from empathy to subjective well-being is significant (*p* < 0.01), while the other paths are significant (*p* < 0.001).

**TABLE 3:** Multiple mediation model fitness index (*n* = 586).

Model	χ^2^	*df*	χ^2^/*df*	*RMSEA*	*CFI*	*TLI*	*SRMR*
M_0_	74.215	22	3.373	0.064	0.977	0.962	0.036
M_1_	316.413	22	11.300	0.066	0.814	0.695	0.098
M_2_	134.686	22	6.122	0.093	0.950	0.918	0.053

Table 3 lists the fitting indices for the three structural models. Hypothesis model M_0_ fits well with the actual data, which indicates that resilience and subjective well-being act as a multiple mediation model between empathy and occupation expectation. Models M_1_ and M_2_ have good fitness, but the fitness is significantly worse than the hypothetical model. Therefore, hypothesis model M_0_ (see Figure 2) is the optimal model, indicating that empathy not only directly predicts the occupation expectation of medical students, but also indirectly affect the level of occupation expectations of medical students through resilience and subjective well-being.

### Multiple Mediated Effects of Resilience and Subjective Well-Being

Bootstrap tests that repeatedly sample model M_0_ 1000 times with a confidence interval of 95% using the M-plus software. From the model point of view, the empathy is intended to affect the occupation expectation of medical students through four paths, including direct path (direct): Empathy → Occupation Expectation, indirect path 1 (indirect1): Empathy → Resilience → Occupation Expectation, indirect path 2 (indirect2): Empathy → Subjective Well-being → Occupation Expectation, indirect path 3 (indirect3): Empathy → Resilience → Subjective Well-being → Occupation Expectation. If all three indirect paths are significant, the multiple mediation effect exists. As can be seen from the data analysis results in Table 4 and Figure 2, the direct path effect value is 0.073 (95% CI: 0.007∼0.217), the indirect path 1 effect value is 0.078 (95% CI: 0.022∼0.134), indirect path 2 effect value is 0.010 (95% CI: 0.005∼0.022), indirect path 3 effect value is 0.022 (95% CI: 0.0604∼0.039), all the confidence intervals do not contain 0, and the mediated effect ratio is 60.109%. According to the above analysis, the model is established. There are multiple mediated effects, that is, empathy can not only have a direct impact on occupation expectation of medical students, but also have an indirect impact through the multiple mediated effect of resilience and subjective well-being on occupation expectation of medical students.

**TABLE 4:** Testing the pathways of the multiple mediation model.

Effect	Path	Effect value	SE	95% confidence interval	
				Lower	Upper
Mediation effect	indirect1	0.204 × 0.381 = 0.078	0.029	0.022	0.134
	indirect2	0.046 × 0.209 = 0.010	0.007	0.005	0.022
	indirect3	0.204 × 0.505 × 0.209 = 0.022	0.009	0.004	0.039
Total mediation effect	indirect1 + indirect2 + indirect3	0.078 + 0.010 + 0.022 = 0.110	0.037	0.037	0.181
Direct effect	Direct	0.073	0.073	0.007	0.217

## Discussion

From the theoretical perspective of multi-factor integration, the study reveals the multiple mediated effects of resilience and subjective well-being in the process of empathy on occupation expectation of medical students and explores the mechanism of improving the level of occupation expectation by empathy ability. Theoretically, the study not only clarifies the question of how empathy “affect” the occupation expectation of medical students (the multiple mediated effects of resilience and subjective well-being), but also responds to the mechanism of action between two mediated variables (the positive prediction of resilience to subjective well-being) and the question of who plays a major role in multiple mediation. It not only enriches the research content of medical psychology, but also can provide guidance for the exploration and development of the framework in action and the integrated factor-process model of resilience and expectation theory of subjective well-being. In reality, the results of the study provide ideas for deepening the study of the relationship between empathy and individual occupational psychology, guiding medical students to form reasonable and firm occupation expectation, and reducing the turnover rate of medical students to promote the development of medical and health undertakings.

### Direct Impact

The results show that empathy has a significant direct effect on medical students’ occupation expectation, indicating that a high degree of medical students’ ability to empathy correlates with a high level of occupation expectation, which is basically consistent with previous research (Kestenbaum et al., 1989; Shanafelt et al., 2005; Sherman and Cramer, 2005; Neumann et al., 2011; Qi et al., 2011; Batt Rawden et al., 2013; Li et al., 2016; Mao, 2019; Ma et al., 2020). This consistency confirms that medical students with strong empathy can establish and develop good doctor-patient relationship with patients in medical practice, help to form a warm, tolerant and understanding character, enhance professional feelings for medical work and obtain higher working satisfaction (Xie et al., 2017), thus improve their value judgment and occupation expectation of the doctor’s profession. On the other hand, medical students with weak empathy are more susceptible to negative events in medical activities, and more difficult to experience and feel the physical pain and psychological burden of patients, acquiring lower sense of accomplishment and getting less occupation expectation.

### Mediating Role of Resilience

Empathy have a positive effect on occupation expectation of medical students through resilience, which is reflected in the positive prediction of resilience, while resilience is also positive prediction of occupation expectation, which is consistent with previous research results (Kinman and Grant, 2010; Matos et al., 2010; Zhang and Jin, 2010; Wang and Song, 2011; Scali et al., 2012; Zhao K. et al., 2013; Morice-Ramat et al., 2018; He et al., 2019) and supports the framework in action and the integrated factor-process model of resilience. Therefore, the individuals’ ability to empathy can improve the perception and relationship satisfaction of social support (Shi et al., 2021), thus they can have a strong adaptability and psychological resistance to pressure, have a high level of resilience, and can actively regulate negative emotions, maintain an optimistic attitude, and keep a good state of the body in the face of difficulties (Campbell Sills and Stein, 2007). Therefore, it is very important to improve the level of occupation expectation by cultivating resilience.

### Mediating Role of Subjective Well-Being

Empathy have a positive effect on occupation expectation of medical students through subjective well-being, which is reflected in the positive prediction of subjective well-being, and subjective well-being has a positive effect on occupation expectation of medical students, which is consistent with previous research (Gruhn et al., 2008; Wei et al., 2011; Wang and Zhang, 2012; Liu et al., 2014; Bourgault et al., 2015; Fang, 2017; Xiao, 2017). Subjective well-being has a role to play in conveying between empathy and occupation expectation. The medical students with high empathy are more likely to be aware of their contributions and roles in helping patients in medical practice activities and therefore more likely to achieve their pre-set career value goals and job satisfaction (Diener and Fujita, 1995; Chen and Zhou, 2003), thereby enhancing their occupation expectation. In addition, as can be found from Table 3, although this mediating effect is significant, the effect value of empathy on subjective well-being (0.046) is much smaller than the effect value of subjective well-being on occupation expectation (0.209), so when we pay more attention to the effect of empathy on subjective well-being, we should concentrate on the effect of subjective well-being on occupation expectation, and put the improvement of subjective well-being of medical students as the focus of the problem of cultivating occupation expectation.

### Sequential Mediating Effect of Resilience and Subjective Well-Being

Findings of the current study validates the hypothesis that empathy have an indirect effect on occupation expectation of medical students through the sequentially mediated effect of resilience and subjective well-being. Medical students with higher ability of empathy feel more social support from family and peers, as well as affirmation and motivation from patients, gain more internal resources to resist the negative environment, thus establishing better psychological resilience, which in turn helps students to look more positively at the impact of the environment, more optimistic about the problems in life, obtain full subjective well-being, and enhance the ability of medical students to cope with job burnout, thus forming a higher level of professional identity and occupation expectation (Huang et al., 2016). This result reveals the close relationship between resilience and subjective well-being, which can be explained by the integrated factor-process model of resilience. In the process of psychological resilience, individuals through dangerous/adversity gradually meet stress coping skills to help them rebound from stress to achieve resilience re-engineering, so as to achieve positive development results, such as acquirement of subjective well-being and the establishment of good occupation expectation. And the negative result is poor adaptation, decreased subjective well-being and lower occupation expectations (Xi et al., 2013). Medical students in medical practice activities will encounter pressures and challenges such as medical contradictions, patient death and job burnout, if the pressure and challenge are not balanced by the external social environment or psychological resilience factors, it will lead to the broken or destroy of dynamic balance of the individual internal system. If there are protective factors such as high psychological resilience, it can promote the individual disorder to ease or eliminate, and then enhance subjective well-being and establish occupation expectation (Kumpfer and Bluth, 2004; Xiao, 2017). In addition, the results of this study also show that the indirect effect of resilience (0.078) is greater than subjective well-being (0.010) in the influence of empathy on the occupation expectation of medical students, which suggests that resilience, as a near-term self-regulating factor affecting the occupation expectation of medical students, plays a greater role than subjective well-being as a far-term cognitive factor. Above conclusion reveals that college educators should focus on the formation and development of resilience of medical students, actively seeking all kinds of interventions to promote the improvement of their resilience level and establish good occupation expectation. On the other hand, we should also pay attention to the negative effect of low level of resilience on medical students to obtain subjective well-being. It is worth noting that the improvement of the medical environment in which doctors live in reality is still in the long-term stage of development, so it is conducive for medical students to establish a high level of psychological resilience, which can help them obtain a higher level of subjective well-being in the current professional environment, thus forming good occupation expectation and promoting the development of medical and health services. Based on the above, medical colleges should fully consider the role of protective factors when cultivating and enhancing the occupation expectation of clinical medical students. Strengthening the intervention of emotional factors (empathy), self-regulating ability (psychological toughness) and cognitive factors (subjective well-being) is an important way to effectively establish professional values, improve occupation expectation of medical students and reduce the turnover rate of medical students.

### Limitations and Revelations of Research

In general, our outcomes were consistent with those of previous studies and fully verified their conclusions, reinforcing the authenticity and credibility of the present study. Nevertheless, the present study had several limitations. Firstly, due to space and time constraints, this study adopts the design of cross-sectional research. Although previous studies have provided a basis for this study, it is still difficult to determine the causal relationship between variables. Future research can use longitudinal or experimental research methods to further verify the results of this study. Secondly, under the multiple mediation model, resilience and subjective well-being only partially mediate the link between empathy and occupation expectation of medical students, suggesting the presence of other mediators. To intervene and promote occupation expectation of medical students, future studies should explore other possible mediators such as family environment, peer support, overall income level of the doctor group and social doctor-patient relationship to explain the mechanisms involved in the impact of empathy on occupation expectation.

## Data Availability Statement

The datasets presented in this study can be found in online repositories. The names of the repository/repositories and accession number(s) can be found in the article/Supplementary Material.

## Ethics Statement

The studies involving human participants were reviewed and approved by the questionnaire and methodology for this study was approved by the Human Research Ethics Committee of the Kunming Medical University (Ethics Approval Number: 2021kmykdx6f01). The patients/participants provided their written informed consent to participate in this study. Written informed consent was obtained from the individual(s) for the publication of any potentially identifiable images or data included in this article.

## Author Contributions

WW, YZ, and ZT: research idea and study design. QQ, XC, SL, and ZG: data collection. WW, LY, XM, and YL: data analysis and manuscript writing. ZL, XY, YC, and QL: supervision, project administration, and funding acquisition. All authors contributed to the article and approved the submitted version.

## Conflict of Interest

The authors declare that the research was conducted in the absence of any commercial or financial relationships that could be construed as a potential conflict of interest.

## Publisher’s Note

All claims expressed in this article are solely those of the authors and do not necessarily represent those of their affiliated organizations, or those of the publisher, the editors and the reviewers. Any product that may be evaluated in this article, or claim that may be made by its manufacturer, is not guaranteed or endorsed by the publisher.
