# Supplementary material for: Relationship Between Medical Students’ Empathy and Occupation Expectation: Mediating Roles of Resilience and Subjective Well-Being
Source: Front Psychol. 2021 Sep 27;12:708342. doi: 10.3389/fpsyg.2021.708342 (PMC8503271; doi:10.3389/fpsyg.2021.708342)
Supplement: Supplementary file 2 [file Data_Sheet_2.PDF]

大学生校园生活调查问卷

亲爱的同学：

您好！我们是大学生创新计划项目组的成员，正在进行关于大学生校园生活现状的调查。现需要您的帮助和参与。

下面是一套调查问卷，主要涉及你在学校的行为表现和感受。大部分题目只需要您在选项上打“√”就可以。这些问卷只用于科学研究，除研究者外，没有人会看到您的答案，答案无对错之分，请您按照真实情况填写。谢谢您的支持与合作！

一、您的基本情况：

请您如实填写下列基本情况：(在适合您的答案后打“√”或填写适当的答案)

1.学号：__________ 2.院系：______学院______专业_____年级

3.性别：男（ ）女（ ） 4.是否学生干部：是（ ）否（ ）

5.是否独生子女：是（ ）否（ ） 6.出生年月：______年___月

7.您的总体学习成绩在班里

1 （ ） 2 （ ） 3 （ ） 4 （ ） 5 （ ）

一点也不好 比较差 一般 比较好 很好

8.您父亲的文化程度：

①小学或小学以下（ ） ②初中（含初中未毕业）（ ） ③高中或中专（含高中未毕业）（ ）

④大专（含夜大、电大）（ ） ⑤大学本科（ ） ⑥研究生（硕士或博士）（ ）

9.您父亲目前从事的职业：

（ ）各级政府部门、企事业单位、党政机关和公众团体的领导者

（ ）专业技术人员（教师、医生、工程技术人员、作家等专业技术人员）

（ ）职员（从事一般性事务工作的人员） （ ）商务人员 （ ）第三产业服务人员

（ ）产业工人 （ ）从事农林牧渔业的劳动者 （ ）家庭主妇

（ ）学生 （ ）私营企业主 （ ）失业 （ ）离退休 （ ）其他行业

10.您母亲的文化程度：

①小学或小学以下（ ） ②初中（含初中未毕业）（ ） ③高中或中专（含高中未毕业）（ ）

④大专（含夜大、电大）（ ） ⑤大学本科（ ） ⑥研究生（硕士或博士）（ ）

11.您母亲目前从事的职业：

（ ）各级政府部门、企事业单位、党政机关和公众团体的领导者

（ ）专业技术人员（教师、医生、工程技术人员、作家等专业技术人员）

（ ）职员（从事一般性事务工作的人员） （ ）商务人员 （ ）第三产业服务人员

（ ）产业工人 （ ）从事农林牧渔业的劳动者 （ ）家庭主妇

（ ）学生 （ ）私营企业主 （ ）失业 （ ）离退休 （ ）其他行业

12.您父母的月总收入：

① 2000元以下（ ） ② 2000—4000元（ ） ③ 4000元—6000元（ ）

④ 6000元—8000元（ ） ⑤ 8000元—10,000元（ ） ⑥ 10,000元—12,000元（ ）

⑦ 12,000元—14,000元（ ） ⑧ 14,000元—16,000元（ ） ⑨ 16,000元以上（ ）

二、下面列出了一些关于您对于个人未来所从事职业的期望的句子。请仔细阅读每一个句子，并根据自己最近一段时间内的实际情况，在每一描述的相应数字上打“√”。

题号	项目	不太重要	一般	有些重要	重要	很重要
1	收入高	1	2	3	4	5

题号	项目	不太重要	一般	有些重要	重要	很重要
2	福利好	1	2	3	4	5
3	职业稳定	1	2	3	4	5
4	能提供受教育的机会	1	2	3	4	5
5	有出国机会	1	2	3	4	5
6	有较高的社会地位	1	2	3	4	5
7	能发挥自己的才能	1	2	3	4	5
8	提供医疗、养老、住房公积金	1	2	3	4	5
9	职业环境优雅	1	2	3	4	5
10	符合兴趣爱好	1	2	3	4	5
11	机会均等，公平竞争	1	2	3	4	5
12	晋升机会多	1	2	3	4	5
13	单位知名度高	1	2	3	4	5
14	单位规模大	1	2	3	4	5
15	能学以致用	1	2	3	4	5
16	交通便利，信息通畅	1	2	3	4	5
17	自主性大，不受拘束	1	2	3	4	5
18	工作有挑战性	1	2	3	4	5
19	容易成名成家	1	2	3	4	5
20	单位级别高	1	2	3	4	5
21	单位在大城市	1	2	3	4	5

三、请根据过去一个月您的情况，选出符合自己的选项，在每一描述的相应数字上打“√”。

题号	项目	从来不	很少	有时	经常	一直如此
1	我能适应变化	0	1	2	3	4
2	我有亲密、安全的关系	0	1	2	3	4
3	有时，命运或上帝能帮忙	0	1	2	3	4
4	无论发生什么我都能应付	0	1	2	3	4
5	过去的成功让我有信心面对挑战	0	1	2	3	4
6	我能看到事情幽默的一面	0	1	2	3	4
7	应对压力使我感到有力量	0	1	2	3	4
8	经历艰难或疾病后，我往往会很快恢复	0	1	2	3	4
9	事情发生总是有原因的	0	1	2	3	4
10	无论结果怎样，我都会尽自己最大努力	0	1	2	3	4
11	我能实现自己的目标	0	1	2	3	4
12	当事情看起来没什么希望时，我不会轻易放弃	0	1	2	3	4
13	我知道去哪里寻求帮助	0	1	2	3	4
14	在压力下，我能够集中注意力并清晰思考	0	1	2	3	4
15	我喜欢在解决问题时起带头作用	0	1	2	3	4
16	我不会因失败而气馁	0	1	2	3	4
17	我认为自己是个强有力的人	0	1	2	3	4
18	我能做出不寻常的或艰难的决定	0	1	2	3	4

题号	项目	从来不	很少	有时	经常	一直如此
19	我能处理不快乐的情绪	0	1	2	3	4
20	我不得不按照预感行事	0	1	2	3	4
21	我有强烈的目的感	0	1	2	3	4
22	我感觉能掌控自己的生活	0	1	2	3	4
23	我喜欢挑战	0	1	2	3	4
24	我努力工作以达到目标	0	1	2	3	4
25	我对自己的成绩感到骄傲	0	1	2	3	4

四、阅读每一个条目，请根据您的实际情况进行评价，在每一描述的相应数字上打“√”。

题号	项目	完全不同意	基本不同意	不确定	基本同意	完全同意
1	朋友的情绪对我影响不大	1	2	3	4	5
2	我很容易受别人情绪的感染	1	2	3	4	5
3	朋友受到惊吓时，我很难察觉到	1	2	3	4	5
4	看到有人哭泣时，我不会难过	1	2	3	4	5
5	他人的情绪根本不会对我造成任何干扰	1	2	3	4	5
6	当某人情绪低落时，我一般能够察觉到	1	2	3	4	5
7	当朋友受到惊吓时，我一般能够察觉到	1	2	3	4	5
8	在看到电视或电影中的悲伤情景时，我常常会随之感伤	1	2	3	4	5
9	一般在人们诉说自己的心情之前，我便能觉察到他们的情绪	1	2	3	4	5
10	看到他人被激怒时，我的情绪不会受到影响	1	2	3	4	5
11	当他人高兴时，我一般能觉察到	1	2	3	4	5
12	我能很快意识到朋友生气了	1	2	3	4	5
13	我常常会卷入朋友的情绪中去	1	2	3	4	5
14	朋友的低落情绪对我没什么影响	1	2	3	4	5
15	我常常觉察不到朋友的情绪感受	1	2	3	4	5
16	当朋友高兴时，我很难觉察到	1	2	3	4	5

五、阅读每一个条目，请根据您的实际情况进行评价，在每一描述的相应数字上打“√”。

题号	项目	强烈反对	反对	有点反对	既不赞成也不反对	有点赞成	赞成	极力赞成
1	我的生活在大多数方面都接近于我的理想	1	2	3	4	5	6	7
2	我的生活条件很好	1	2	3	4	5	6	7
3	我对我的生活很满意	1	2	3	4	5	6	7
4	到现在为止，我已经得到了在生活中我想要得到的重要东西	1	2	3	4	5	6	7
5	如果我能再活一次，我基本上不会做任何改变	1	2	3	4	5	6	7

六、阅读每一个条目，请根据您在过去一个星期的实际情况进行评价，在每一描述的相应数字上打“√”。（变化趋势：由无到有，由弱到强）

题号	项目	根本没有			一半的时间			所有时间
1	愉快	1	2	3	4	5	6	7
2	不愉快	1	2	3	4	5	6	7
3	幸福	1	2	3	4	5	6	7
4	振奋	1	2	3	4	5	6	7
5	难过	1	2	3	4	5	6	7
6	生气	1	2	3	4	5	6	7
7	自豪	1	2	3	4	5	6	7
8	感激	1	2	3	4	5	6	7
9	爱	1	2	3	4	5	6	7
10	负罪感	1	2	3	4	5	6	7
11	羞愧	1	2	3	4	5	6	7
12	担心	1	2	3	4	5	6	7
13	压力	1	2	3	4	5	6	7
14	忌妒	1	2	3	4	5	6	7

七、以下每题有五个选项，按照符合程度排列，请根据您的实际情况，选择出一个与您的情况最为符合的选项。

题号	项目	完全不符合	比较不符合	不确定	比较符合	完全符合
1	我有自己的学习方法和计划，并能付诸实践	1	2	3	4	5
2	我觉得所学知识毫无用处	1	2	3	4	5
3	专业知识的掌握对我来说很容易	1	2	3	4	5
4	清早起来，想到要面对一天的学习，便感到很疲倦	1	2	3	4	5
5	我很难对学习保持长久的热情	1	2	3	4	5
6	学习时，我能够冷静地处理自己情绪上的问题	1	2	3	4	5
7	一整天学习下来我感到精疲力尽	1	2	3	4	5
8	到目前为止，大学学习使我的能力得到充分展示	1	2	3	4	5
9	我对学习感到厌倦	1	2	3	4	5
10	我课后很少学习	1	2	3	4	5
11	我能胜任大学的课程	1	2	3	4	5
12	我学习时经常打瞌睡	1	2	3	4	5
13	我对我的专业很感兴趣	1	2	3	4	5
14	在学习上我觉得我的耐心还不够	1	2	3	4	5
15	对我来说拿到学士学位很容易	1	2	3	4	5
16	只有考试时我才会读书	1	2	3	4	5
17	我想学习，但我感到学习很枯燥	1	2	3	4	5
18	学习时我精力充沛	1	2	3	4	5
19	我很少安排自己的学习时间	1	2	3	4	5
20	考试总是让我厌烦	1	2	3	4	5
